# Supplementary material for: Amino acid catabolite markers for early prognostication of pneumonia in patients with COVID-19
Source: Nat Commun. 2023 Dec 20;14:8469. doi: 10.1038/s41467-023-44266-z (PMC10733290; doi:10.1038/s41467-023-44266-z)
Supplement: Supplementary file 1 — Supplementary information [file 41467_2023_44266_MOESM1_ESM.pdf]

Supplementary Information for

Amino acid catabolite markers for early prognostication of pneumonia in patients with COVID-19

Supplemental Figures

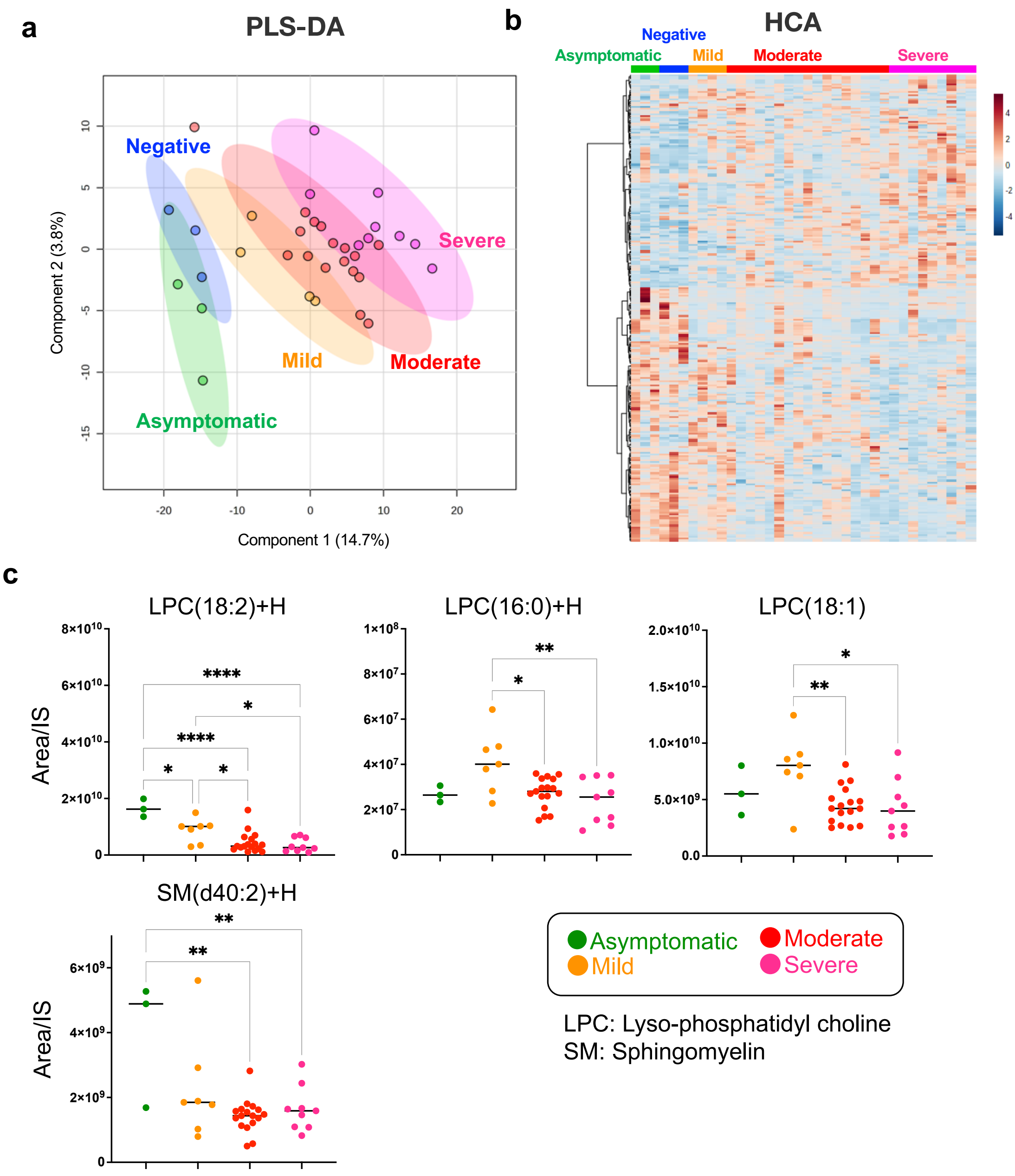

Supplementary Figure 1. Changes in serum lipidome in the early phase of COVID-19 onset

Changes in the serum lipidome of asymptomatic (n=3), mild (n=7), moderate (n=17), and severe (n=9) outcome patients (Cohort-1), collected within 5 days of onset, were evaluated by lipidome analysis. For this purpose, hundreds of phospholipids, including mainly polar phospholipids, nonpolar cholesterol esters, and triacylglycerols, were comprehensively measured by Fourier transform mass spectrometry.

(a) PLS-DA score plots for asymptomatic, mild, moderate, and severe patients in the early stages of COVID-19 development. (b) The summary of lipidome changes by HCA. (c) Quantification of LPC (18:2), LPC (16:0), LPC (18:1), SM (d40:2). Data are expressed as the mean value. Statistical significance was assessed using one-way ANOVA with Tukey's multiple comparisons test. * $p < 0.05$; ** $p < 0.01$; **** $p < 0.0001$.

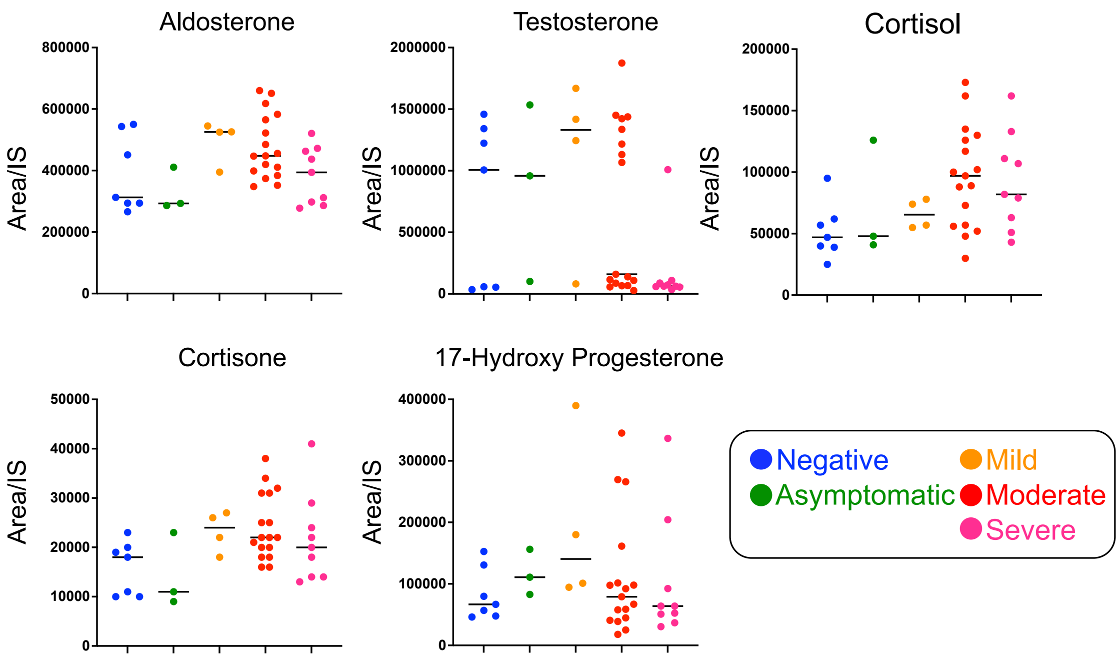

Supplementary Figure 2. Changes in serum steroids in early onset of COVID-19
Changes in serum steroids in SARS-CoV2-negative (n=7), asymptomatic (n=17), mild (n=9), moderate, and severely ill patients (Cohort-1) collected within 5 days of onset of disease were evaluated with a targeted mass spectrometer.

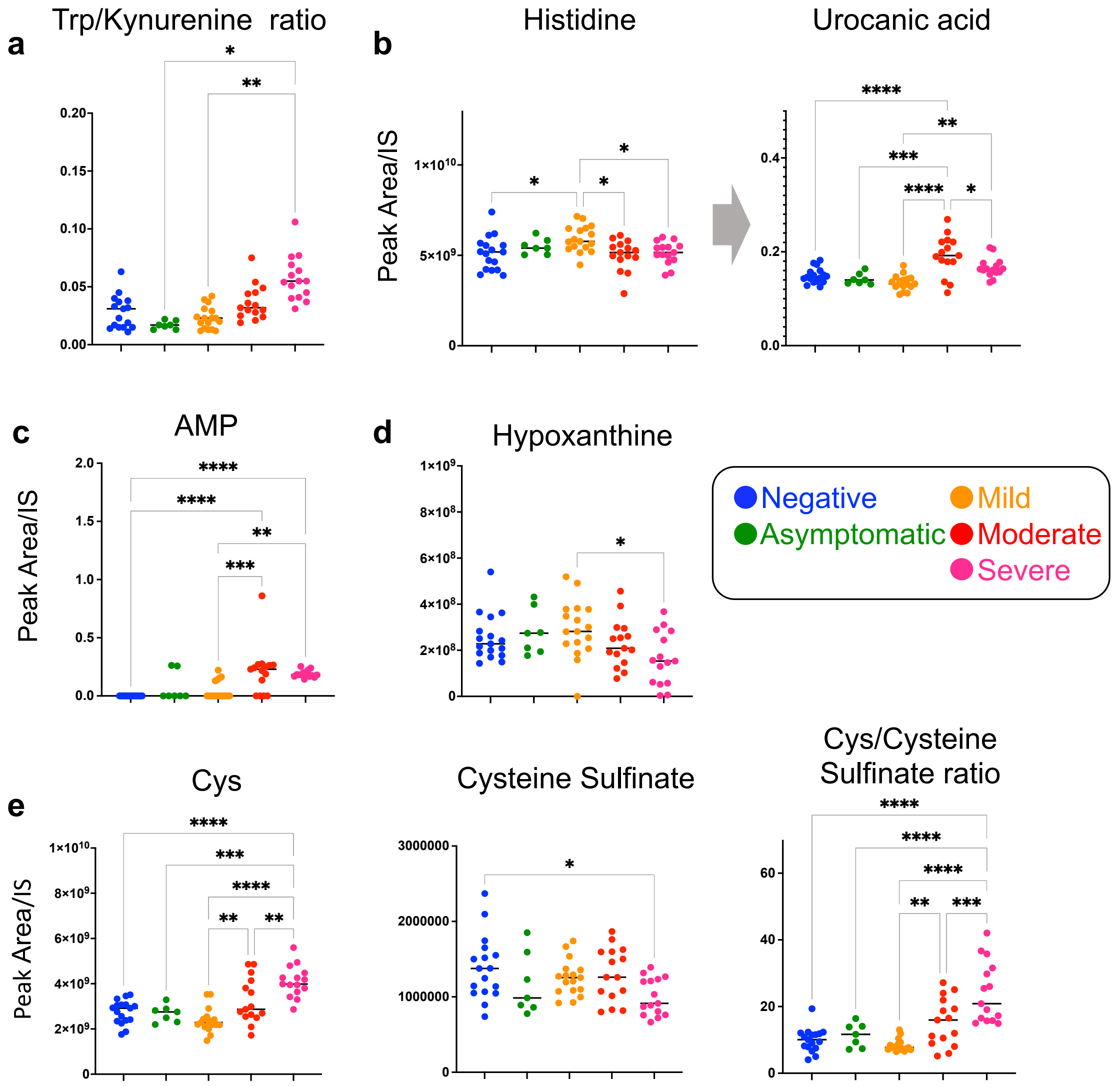

Supplementary Figure 3. Other characteristic changes in serum metabolites induced by COVID-19 early in disease correlate with future severity of pneumonia

Metabolomic analysis of serum from negative (n=17), asymptomatic (n=7), mild (n=17), moderate (n=15), and severe (n=15) outcome patients (Cohort-1 samples) collected within 5 days of disease onset. Trp/Kynurenine ratio (a). Histidine and a deamino catabolite urocaninic acid (b). AMP, a purine nucleotide (c) and hypoxanthine (d), a degradation product of purine nucleotides. Cysteine (e), Cystein sulfinate and cystein sulfinate/Cys ratio. Data are expressed as the mean value. Statistical significance was assessed using one-way ANOVA with Tukey's multiple comparisons test. * $p < 0.05$; ** $p < 0.01$; *** $p < 0.001$; **** $p < 0.0001$.

a

	asymptomatic	mild	moderate	severe	Chi-squared sum	p-value
General malaise (%)	0	5.9	41.2	47.1	11.5	0.009
Dyspnea(%)	0	23.5	29.4	23.5	2.5	0.471
Chest pain(%)	0	5.9	29.4	11.8	5.6	0.135
Neurological/ psychiatric symptoms (%)	14.3	0	5.9	17.6	3.8	0.283

b

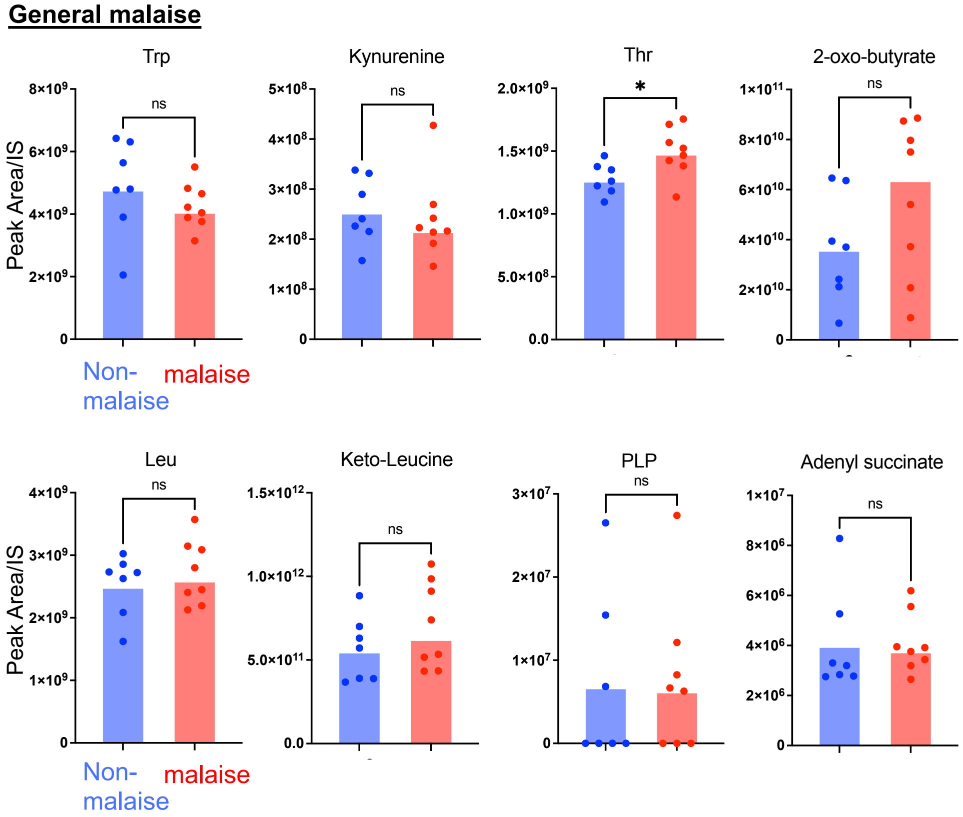

Supplementary Figure 4. The association between serum metabolite levels and superficial symptoms in the acute phase of COVID-19

Patients in Cohort-1 were assessed for the presence of the following superficial symptoms;
1. Neurological/psychiatric symptoms, 2. Chest pain, 3. Dyspnea, 4. General malaise.

Correlation analysis (chi-squared test) between the frequency of occurrence of these symptoms and COVID-19 outcomes showed that only general malaise was significantly correlated (a), suggesting that the occurrence of general malaise was associated with poorer prognosis. The higher viral load in the poor prognosis cases in Cohort-1 of this study (Fig. 3) suggests a possible association with the induction of general malaise. On the other hand, the presence of general malaise was not significantly related to the levels of amino acids and their catabolites which correlated with the prognosis of COVID-19 shown in this study (b). Similar results were confirmed for the presence of dyspnea. Thus, the presence or absence of superficial symptoms does not appear to be reflected in differences in blood metabolite levels early in the course of the disease. Data are expressed as the mean value. Statistical significance was assessed using student-t test. * $p < 0.05$.

a **Summary of comorbidities (Severe outcome)**

**Infectious or non-infectious inflammatory and autoimmune diseases (n=3)**
ulcerative colitis, COPD, tuberculosis,
**Lifestyle-related diseases (n=6):**
Diabetes mellitus, Hypertension, Dyslipidemia.
**No complications or other diseases (n=4)**
**Patients with Malignant tumors(n=2)**

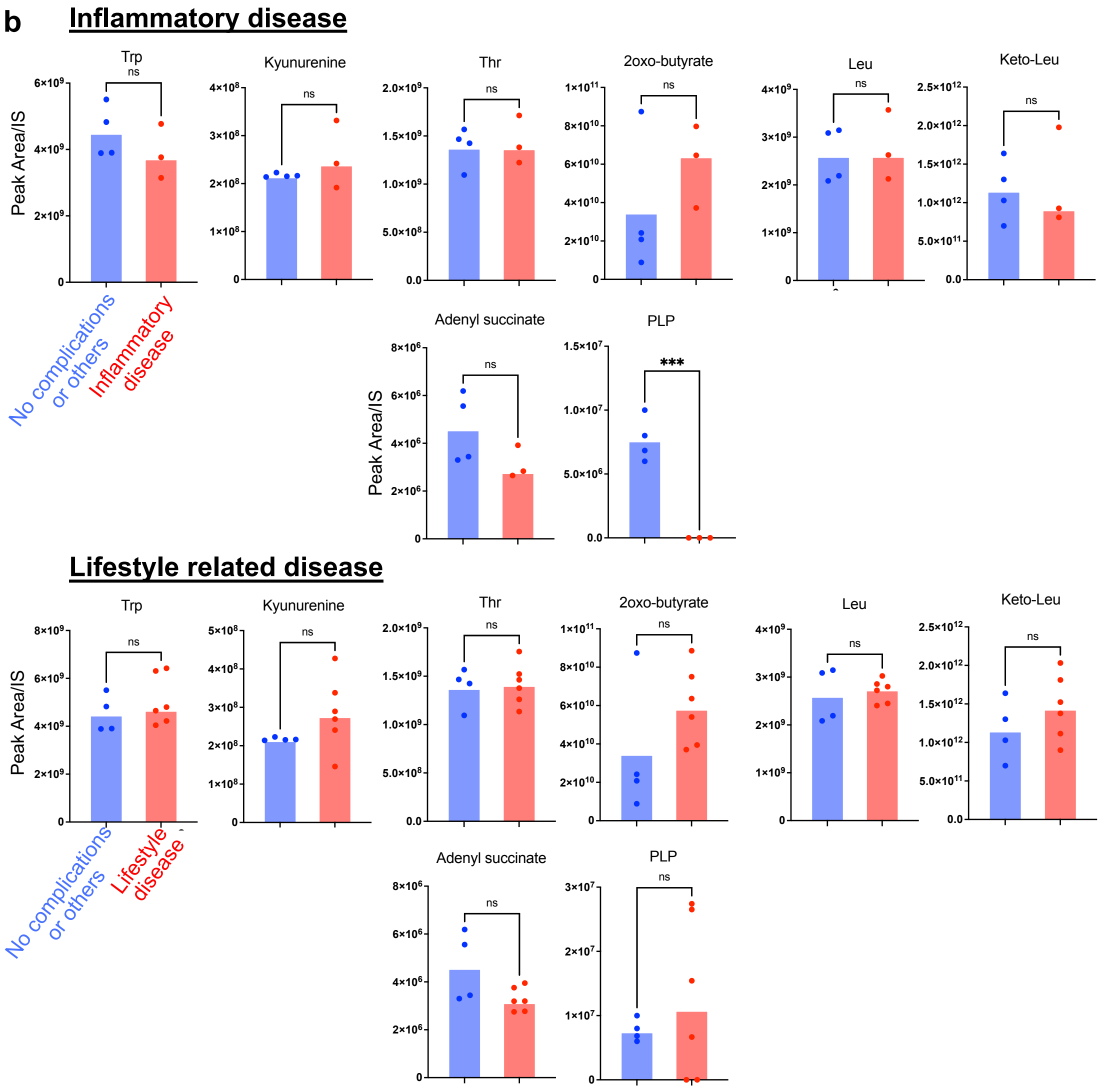

Supplementary Figure 5. The association between serum metabolite levels and comorbidities in the acute phase of COVID-19

We tested whether serum metabolites, which are altered by COVID19 prognosis, are affected by the presence or type of comorbidities. In Cohort-1, most patients with moderate or severe outcomes had some comorbidity. Therefore, we categorized comorbidities (a) and examined the relationship between their presence or absence and serum metabolite concentrations (b): For each of the above complication categories, metabolite levels were compared to the "no complications, other" group.

However, few statistically significant differences were found in metabolites correlated with COVID-19 severity ($p>0.01$).
The only significant difference observed between patients with and without comorbidities was in serum PLP levels. As shown in the text, compared to patients with mild symptoms of COVID-19, patients with severe outcome showed a significant decrease in serum PLP (Fig. 2). Furthermore, among the severe outcome group, patients with inflammatory complications showed a dramatic decrease in PLP levels. This decrease may be due to decreased PLP production due to hepatic dysfunction or increased PLP consumption by inflammatory tissues. In addition, because PLP itself has an inhibitory effect on the inflammasome[1] a marked decrease in PLP may lead to a vicious cycle that exacerbates excessive inflammation and promotes disease severity.
Data are expressed as the mean value. Statistical significance was assessed using student-t test. *** $p < 0.001$.

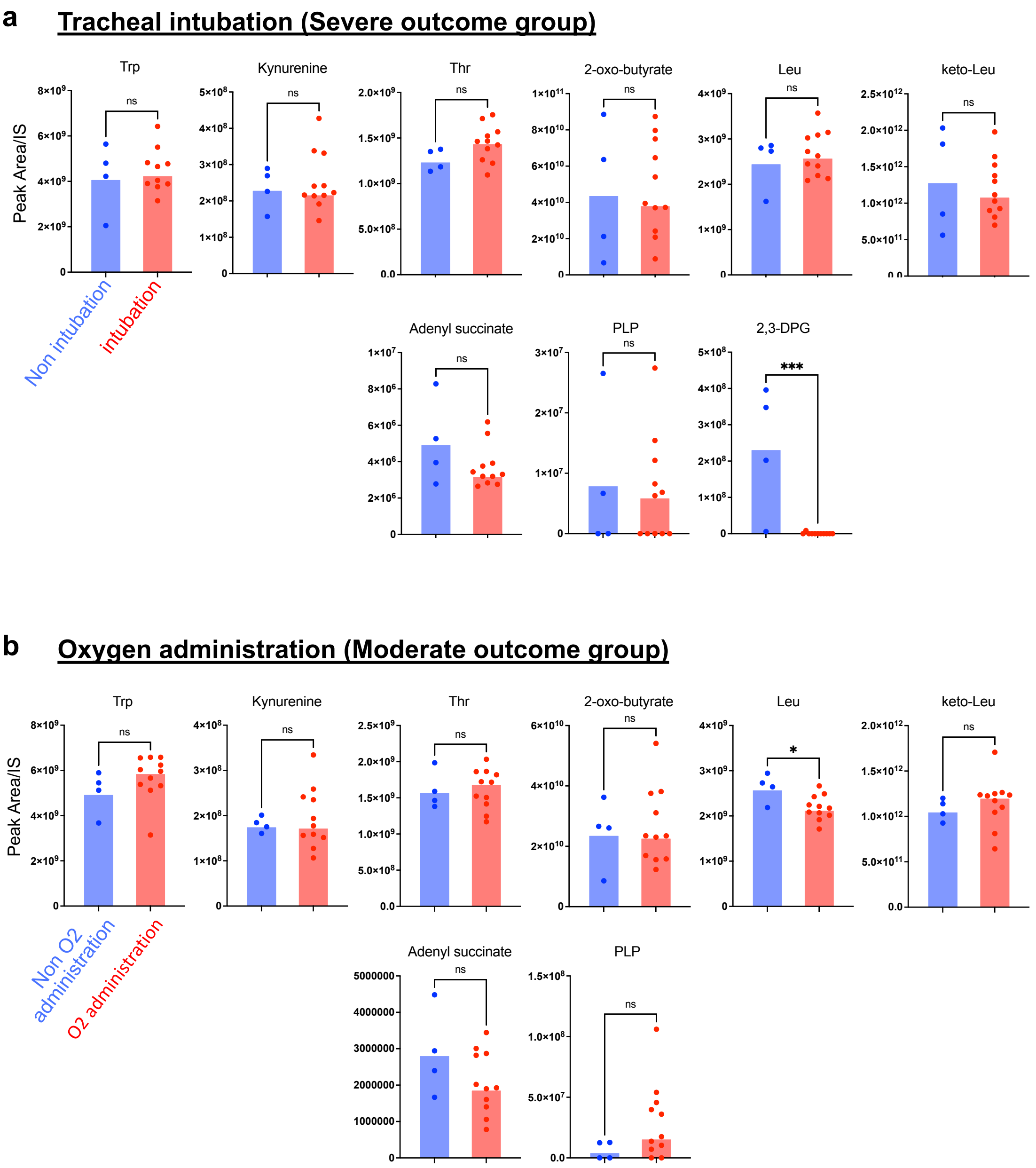

Supplementary Figure 6. The association of serum metabolite levels of COVID-19 in the acute phase with treatment during severe pneumonia

We examined whether the presence or absence of tracheal intubation (a) or (b) in poor prognosis patients impacted the concentration of potential amino acid catabolite indicators for COVID-19 severity. The findings indicated no statistically significant variances ($p>0.01$) among the amino acids and their catabolites discussed in this manuscript, possibly due to the limited sample size ($n=4$ and 10 for non-intubated and intubated patients, respectively). As an example, there were no significant differences in Trp and Thr and their respective catabolites between the oxygenation and airway intubation groups. However, patients who underwent airway intubation had significantly lower serum concentrations of 2,3-diphosphoglyceric acid (2,3-DPG), which is highly concentrated in red blood cells[2]. The lower serum 2,3-DPG concentrations in patients requiring future intubation may be the result of altered metabolism in their red blood cells. Data are expressed as the mean value. Statistical significance was assessed using student-t test. * $p < 0.05$; *** $p < 0.001$.

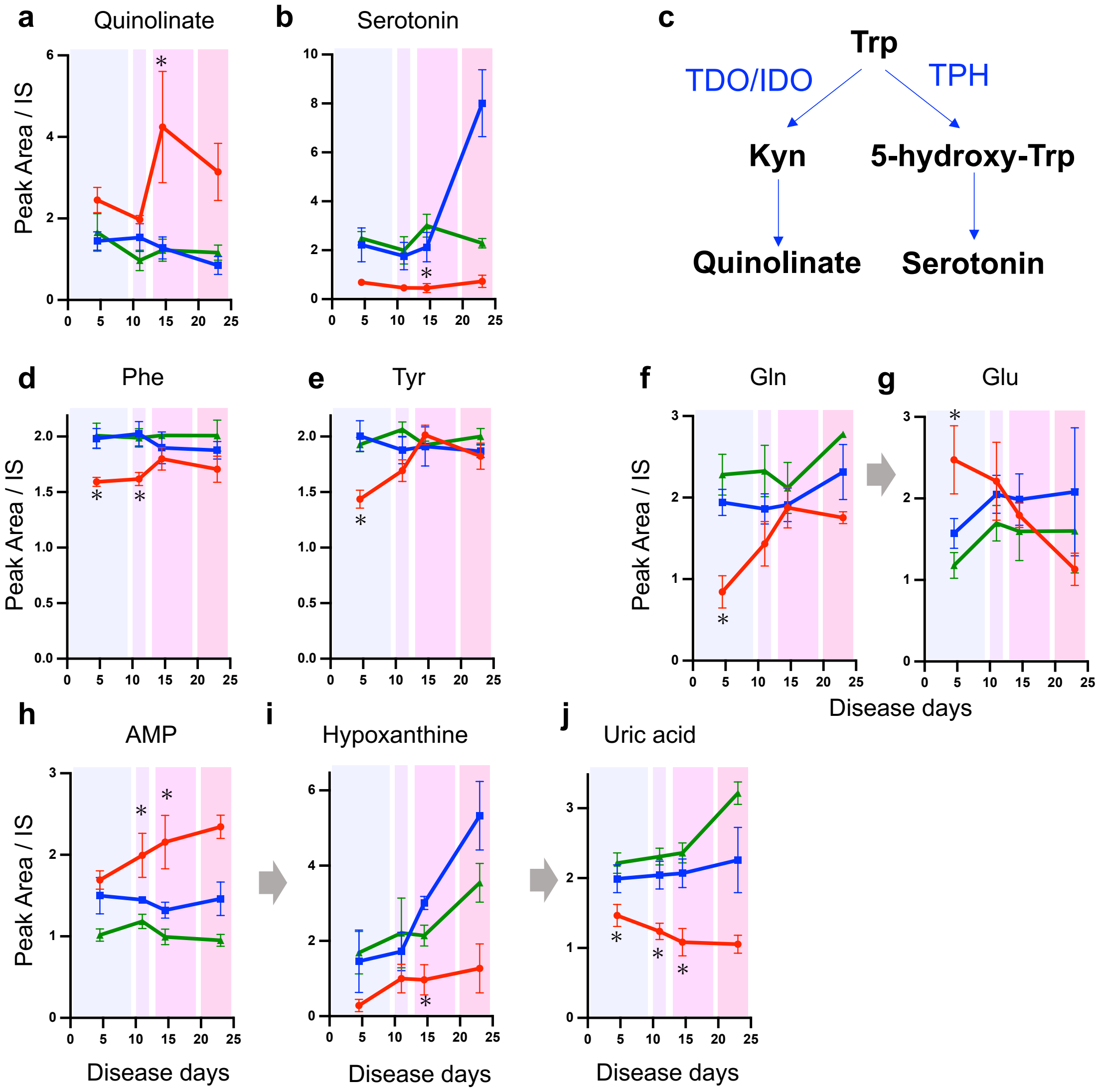

Supplementary Figure 7. Increased serum amino acid deaminocatabolism was specific to the early phase of COVID-19 severe outcome patients

Serum samples from Cohort-2 were used to study changes over time in serum metabolite levels at each of four time points: day 9, days 10-12, days 13-16, and day 20 from the onset date in patients in the severe (n=3-4), moderate (n=2-4), and mild (n=2-4) disease groups. Plotting the time course fluctuations of quinolinic acid (a), serotonin (b), the aromatic amino acids Phe (d) and Tyr (e), Gln (f) and the deamino catabolite Glu (g), AMP (h), hypoxanthine (i) and uric acid (j) levels along the disease day. Diagram of the Trp catabolic pathway (c). Data are expressed as the mean value ± SEM. Statistical significance was assessed using 2-way ANOVA with Tukey's multiple comparisons test. * $p < 0.05$.

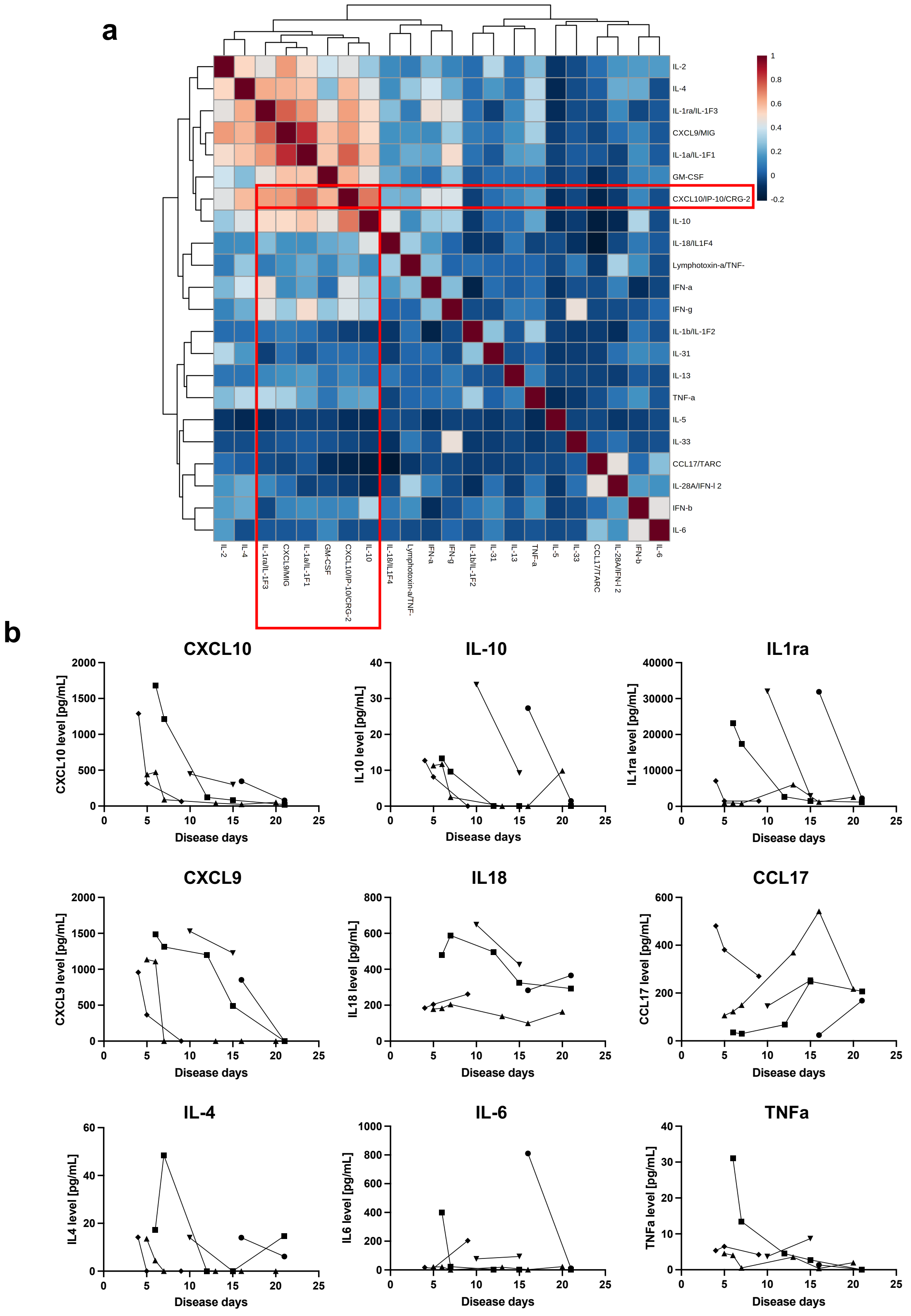

Supplementary Figure 8. Correlation analysis of serum cytokine concentrations and their changes over disease period

(a) Correlation analysis between cytokines in Cohort-1 samples. Correlations between blood

concentrations of cytokines were calculated, with higher correlation coefficients expressed in red and lower ones in blue. Furthermore, cytokines showing similar behavior were classified by hierarchical clustering, and IL10, IL1ra, CXCL9, and IL1a were found to be cytokines with high correlation to CXCL10. (b) In a cytokine panel analysis using serum from Cohort-2 patients with severe outcome, nine cytokines were detected with sufficient sensitivity and presented their temporal behavior at different stages of the disease.

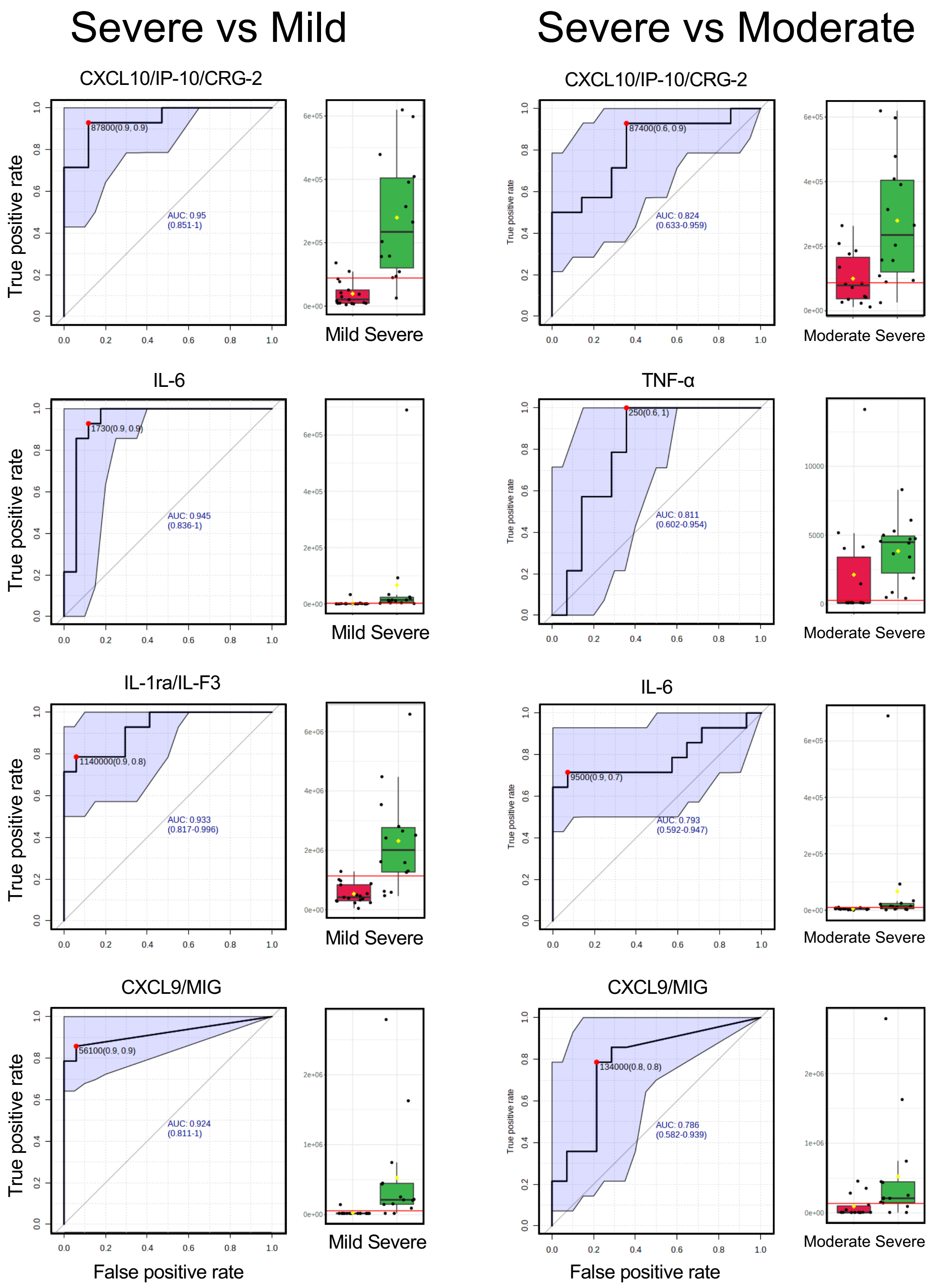

Supplementary Figure 9. Evaluation of prognostic performance of cytokines by ROC analysis
The prognostic performance of Cohort-1 serum samples for discrimination of severe (n=14) and moderate (n=15) disease (left panel) and severe (n=14) and mild (n=16) disease (right panel) was evaluated by ROC analysis. The four ROC plots that showed high performance in each comparison (see also Table 4) are shown.

Name	r2	P-Value
IL-10	-0.29429	0.036062
CXCL10/IP-10	-0.26271	0.062535
IL-6	-0.25554	0.070322
IL-1a/IL-1F1	-0.11873	0.65954
Lymphotoxin	-0.10017	0.4843
IL-18/IL1F4	-0.084194	0.55693
IFN-g	-0.08045	0.57466
IL-1ra/IL-1F3	-0.063193	0.40664
IL-4	-0.059617	0.67773
IL-13	-0.05072	0.72374
CXCL9/MIG	-0.044525	0.75638
TNF-α	-0.0088263	0.96098
CCL17/TARC	0.0096814	0.94624
IL-2	0.022175	0.87725
GM-CSF	0.031692	0.82527
IL-33	0.059807	0.67676
IL-28A/IFN	0.067477	0.63802
IFN-b	0.13489	0.3453
IL-31	0.17172	0.22824
IL-1b/IL-1F2	0.191	0.17941
IL-5	0.19104	0.17931

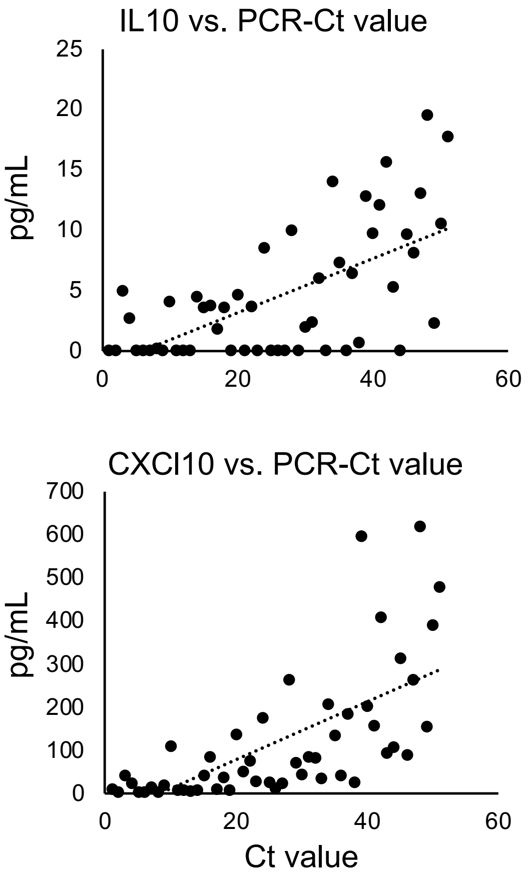

Supplementary Figure 10. Low correlation between serum cytokines and PCR-Ct values compared to the serum metabolites

Two-sided Pearson's correlation analysis of serum cytokine levels and PCR-Ct values using samples from early COVID-19 onset (Cohort-1 samples) (left panel); Correlation plots with cytokine levels (IL10 and CXCL10) on the vertical axis and PCR-Ct values on the horizontal axis are shown (right panel).

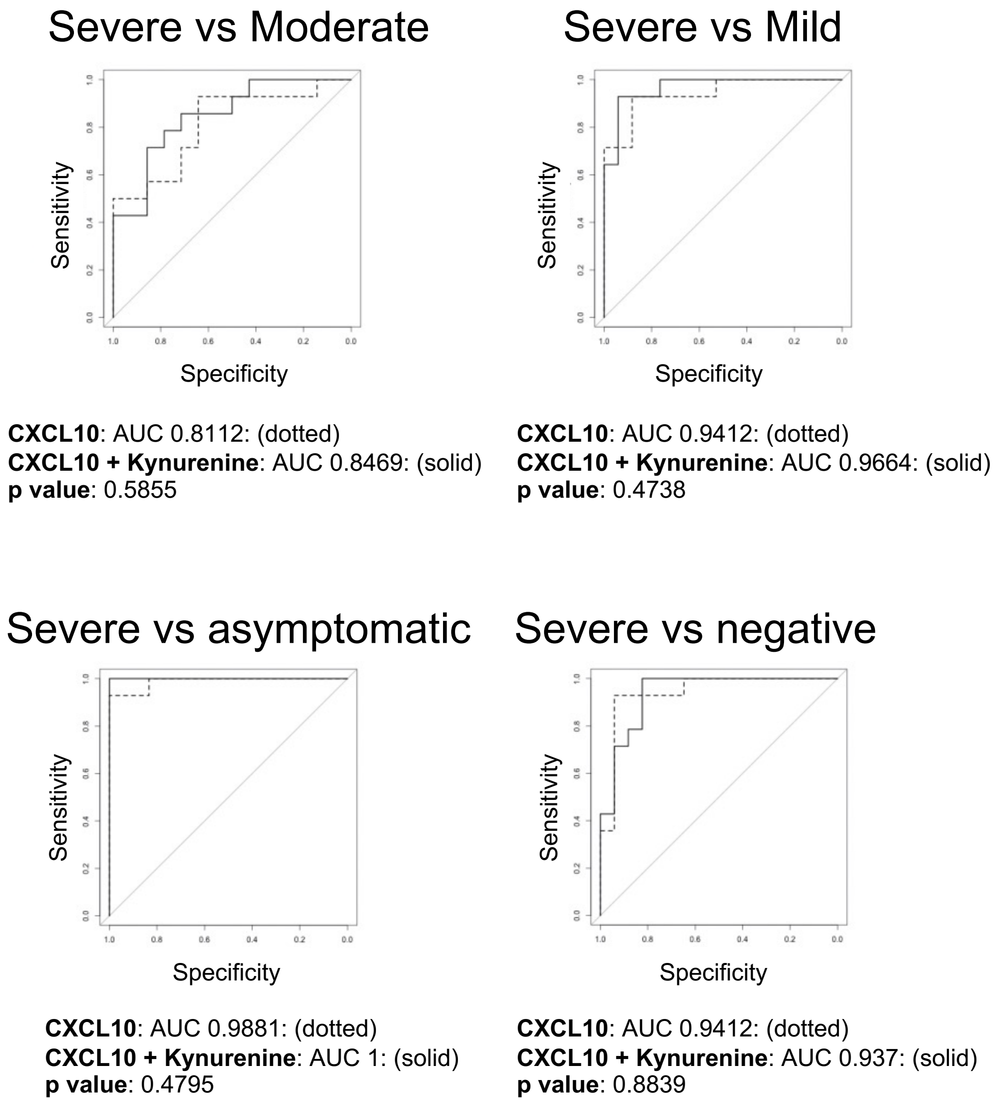

Supplementary Figure 11. Serum kynurenine levels did not improve the prognostic ability of CXCL10 in early COVID-19 onset

ROC analysis evaluated whether a synthetic variable combining serum CXCL10 and kynurenine improved the ability to separate severe from moderate, severe from mild, severe from asymptomatic, and severe from SARS-COV2 negative patients, but did not improve the predictive ability.

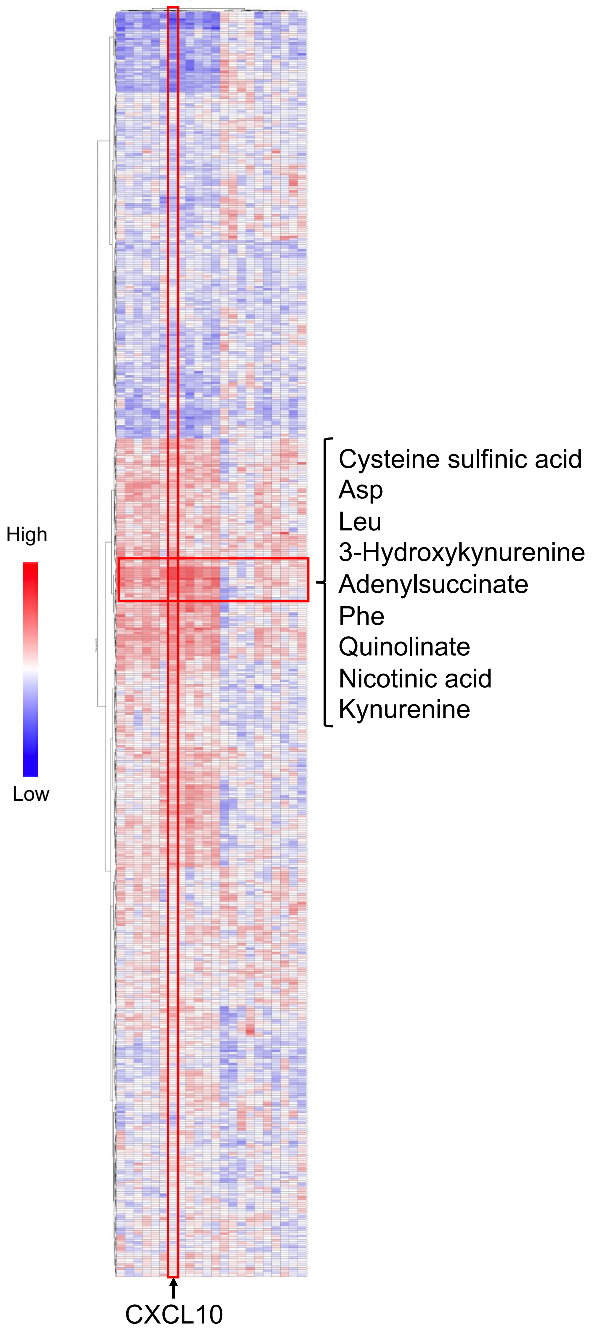

Supplementary Figure 12. Intermediate metabolites of the IDO pathway, BCAA and their catabolites are highly correlated with CXCL10

Correlation analysis of metabolite groups and cytokines in Cohort-1 samples. Each metabolite was placed on the horizontal axis and cytokines on the vertical axis, and the correlation of their blood concentrations was calculated, with high correlation coefficients shown in red and low in blue. In addition, metabolites and cytokines showing similar behavior were classified by hierarchical clustering. Clusters with particularly high correlation coefficients, surrounded by red squares, included CXCL10, Trp catabolites, and BCAA catabolites as metabolite-cytokine combinations.

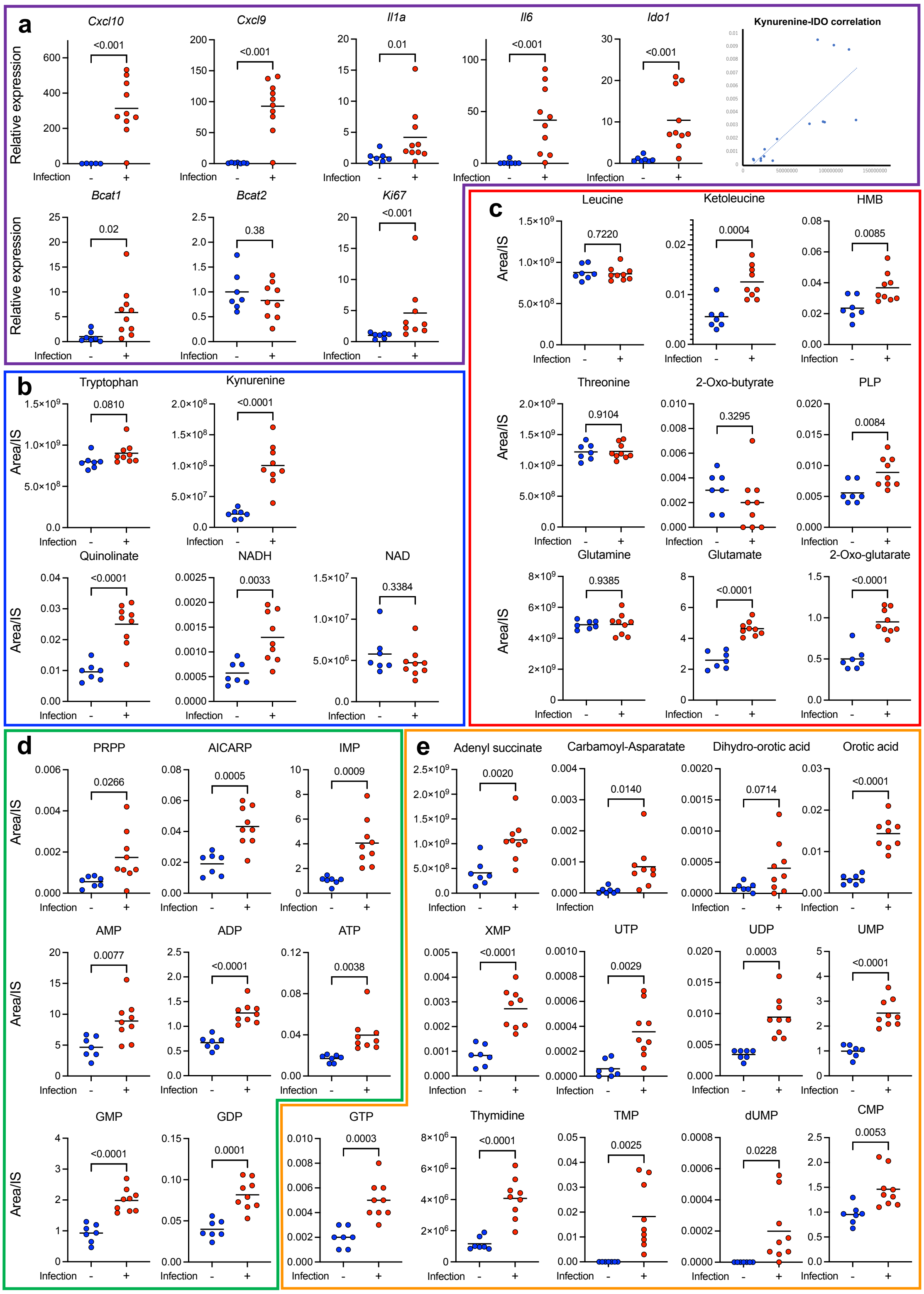

Supplementary Figure 13. Metabolic remodeling with progressive deamination of amino acids and production of de novo nucleotides occurs in lung tissue during the early stages of influenza infection

Mice were infected with influenza and lung tissues were extracted 4 days after infection (n=7-9). The mRNA expression levels of cytokines, chemokines, metabolic enzymes, and Ki67 as a cell

proliferation marker were quantitatively evaluated by qPCR (a). Metabolome profiles of lung tissues of IDO/TDO pathway (b), BCAA and its catabolites (c), Thr and Gln and their catabolites (d, e) and on the *de novo* nucleotide generation pathway. Data are expressed as the mean value. Statistical significance was assessed using student-t test. p- values are shown at the top of the plots.

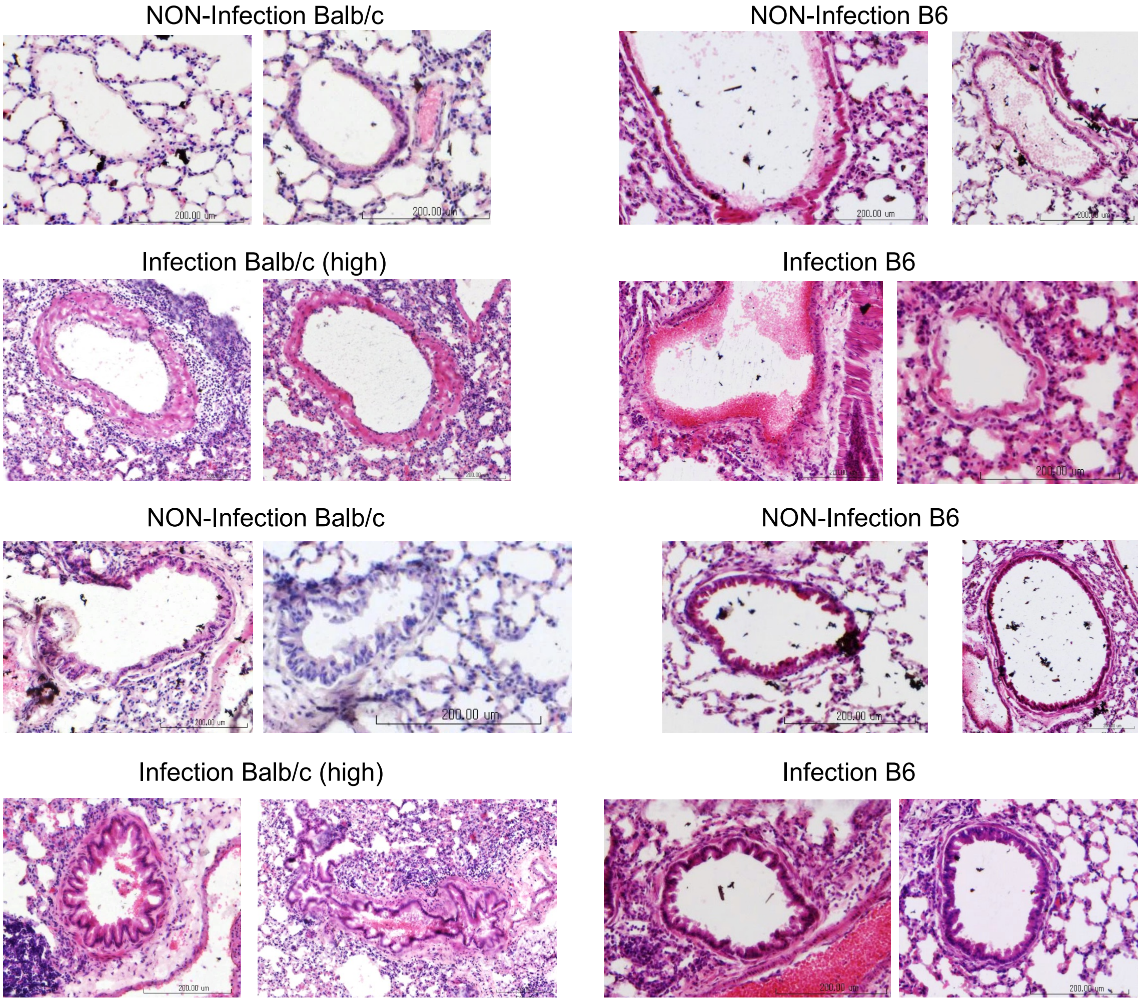

Supplementary Figure 14. Remodeling of vascular smooth muscle and airway epithelium occurs early in the pathogenesis of SARS-COV2-MA10-infected mouse lungs

Lung tissue from B6 strain mice infected with SARS-COV2 was HE stained to compare the pathogenesis of the early stages of infection. Both infection models showed hyperproliferation of vascular smooth muscle (upper panel) and mucosal secretory cells (lower panel) Representative images observed from two individuals are shown.

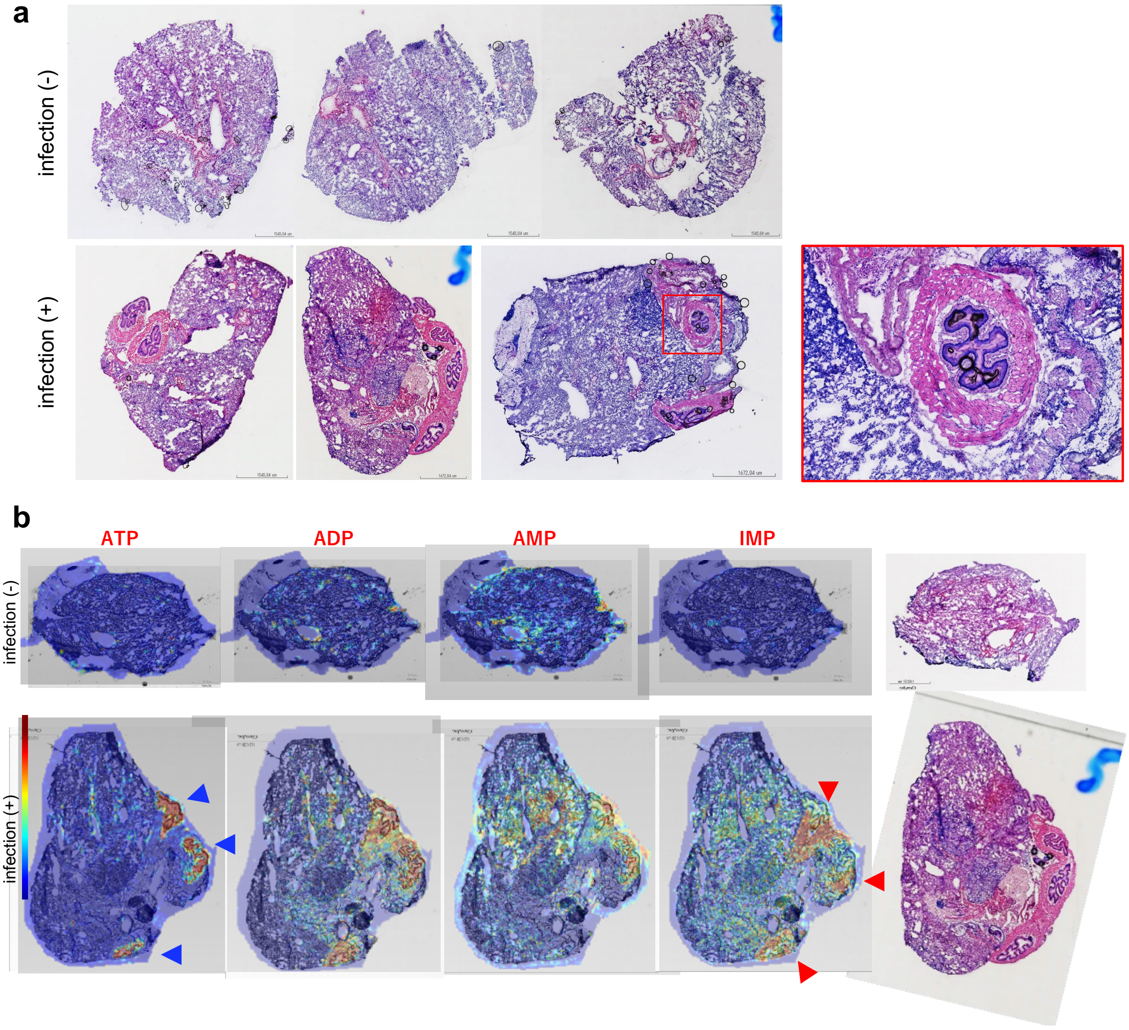

Supplementary Figure 15. Influenza-infected mouse lungs show increased accumulation of nucleotides in hyperproliferated vascular smooth muscle and airway epithelial cells

(a) Lung tissue from influenza infected B6 strain mice were HE stained to compare the pathogenesis of early infection. Hyperproliferation of airway smooth muscle cells and mucosal secretory cells is shown in red square, enlarged image. (b) Imaging MS visualized the localization of IMP, ATP, ADP, and AMP. HE staining is shown on the right. Accumulation of IMP in the thickened airway epithelial cells (blue) and ATP in the hypertrophied orbital smooth muscle tissue (red) are shown with arrow.

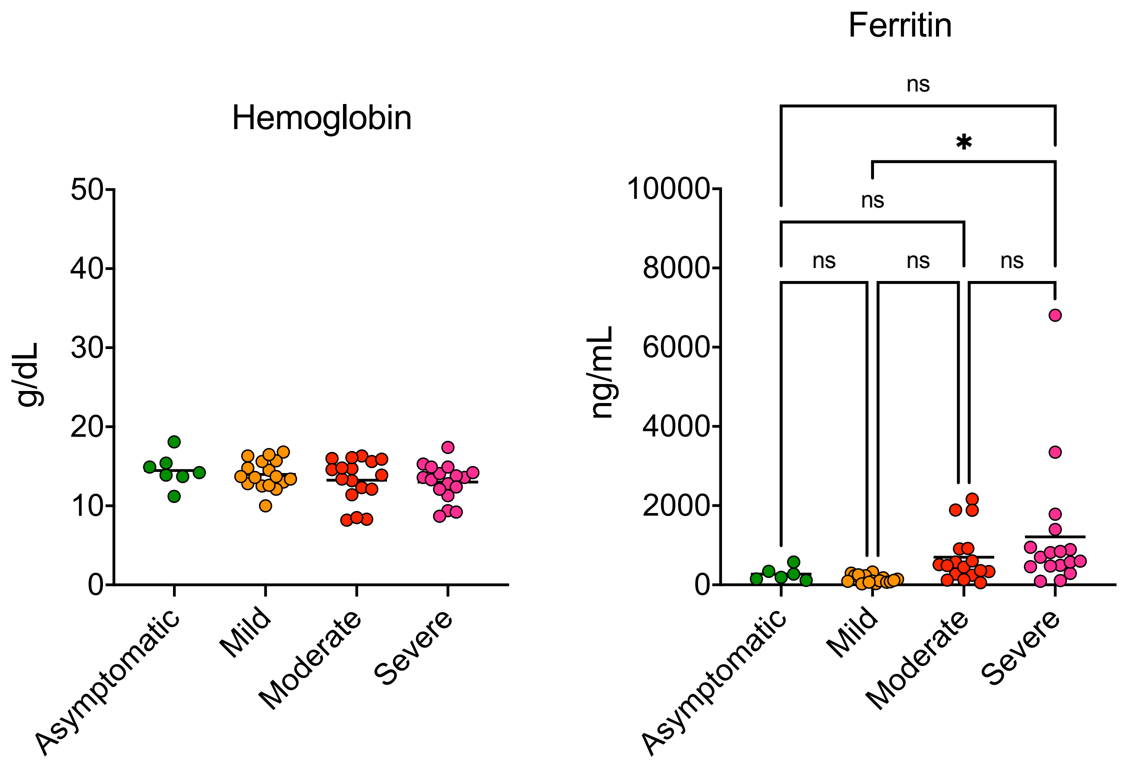

Supplementary Figure 16. Hemoglobin and ferritin levels for Cohort-1
At early onset, contemporaneous with the metabolome measurements, there were no differences in hemoglobin between asymptomatic (n=7), mild (n=17), moderate (n=17), and severe (n=17) groups (a). In contrast, there was a predominant increase in ferritin in the severe disease group (n=17) compared to the mild (n=16) disease group (b). Statistical significance was assessed using one-way ANOVA with Tukey's multiple comparisons test. * $p < 0.05$.

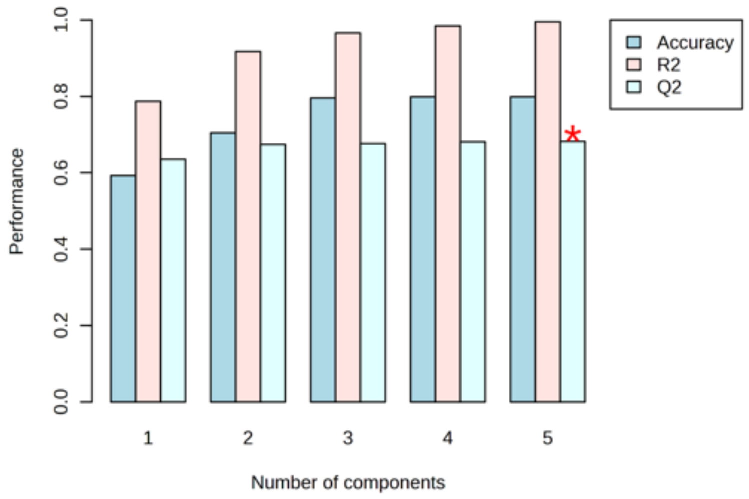

PLS-DA cross validation details

Measure	1 comps	2 comps	3 comps	4 comps	5 comps
Accuracy	0.59303	0.70455	0.79576	0.79909	0.79909
R2	0.78665	0.91758	0.96615	0.98461	0.99485
Q2	0.63571	0.67457	0.67597	0.68082	0.68249

Supplementary Figure 17. PLS-DA cross-validation details for Fig. 1a
Plots obtained by the leave-one-out cross-validation (LOOCV) method applied to partial least squares discriminant analysis (PLS-DA) data. The PLS-DA cross-validation data showed cumulative values of R2 = 0.999 and Q2 = 0.722, indicating good clustering and good discrimination between the groups studied.

Supplemental Tables

Study	MHLW	Age	Sex	Anamnesis	Subjective symptoms	O_2	Intubation	Death
Cohort-1	Negative	-	-	-	-	-	-	-
Cohort-1	Negative	-	-	-	-	-	-	-
Cohort-1	Negative	-	-	-	-	-	-	-
Cohort-1	Negative	-	-	-	-	-	-	-
Cohort-1	Negative	-	-	-	-	-	-	-
Cohort-1	Negative	-	-	-	-	-	-	-
Cohort-1	Negative	-	-	-	-	-	-	-
Cohort-1	Negative	-	-	-	-	-	-	-
Cohort-1	Negative	-	-	-	-	-	-	-
Cohort-1	Negative	-	-	-	-	-	-	-
Cohort-1	Negative	-	-	-	-	-	-	-
Cohort-1	Negative	-	-	-	-	-	-	-
Cohort-1	Negative	-	-	-	-	-	-	-
Cohort-1	Negative	-	-	-	-	-	-	-
Cohort-1	Negative	-	-	-	-	-	-	-
Cohort-1	Negative	-	-	-	-	-	-	-
Cohort-1	Negative	-	-	-	-	-	-	-
Cohort-1	Asymptomatic	50-56	M	-		N	N	N
Cohort-1	Asymptomatic	50-56	F	Atopic dermatitis		N	N	N
Cohort-1	Asymptomatic	60-69	M	-		N	N	N
Cohort-1	Asymptomatic	40-49	M	-	Neurological/psychiatric symptoms	N	N	N
Cohort-1	Asymptomatic	50-56	F	Liver hemangioma		N	N	N
Cohort-1	Asymptomatic	30-39	F	-		N	N	N
Cohort-1	Asymptomatic	70-79	F	-		N	N	N
Cohort-1	Mild	60-69	M	Esophageal cancer		N	N	N
Cohort-1	Mild	30-39	M	-		N	N	N
Cohort-1	Mild	20-29	F	Atopic dermatitis		N	N	N
Cohort-1	Mild	50-56	M	-	General malaise	N	N	N
Cohort-1	Mild	20-29	M	-	Dyspnea	N	N	N
Cohort-1	Mild	30-39	F	-	Dyspnea	N	N	N
Cohort-1	Mild	20-29	F	-		N	N	N
Cohort-1	Mild	20-29	F	-	Dyspnea	N	N	N

Cohort-1	Mild	50-56	F	Dermatomyositis, interstitial pneumonia		N	N	N
Cohort-1	Mild	60-69	M	Hypertension, dyslipidemia, hyperuricemia		N	N	N
Cohort-1	Mild	10-19	F	Osteosarcoma		N	N	N
Cohort-1	Mild	30-39	F	Pregnant women		N	N	N
Cohort-1	Mild	20-29	F	Eating disorders		N	N	N
Cohort-1	Mild	20-29	F	Kallmann's syndrome		N	N	N
Cohort-1	Mild	20-29	M	-		N	N	N
Cohort-1	Mild	20-29	M	-	Dyspnea	N	N	N
Cohort-1	Mild	30-39	M	-	Chest pain	N	N	N
Cohort-1	Moderate	80-89	M	Prostate cancer	General malaise	Y	N	N
Cohort-1	Moderate	80-89	M	Gastric cancer, aortic valve stenosis		N	N	N
Cohort-1	Moderate	80-89	M	MPO-ANCA	Neurological/psychiatric symptoms	Y	N	N
Cohort-1	Moderate	50-59	M	Lung cancer		N	N	N
Cohort-1	Moderate	60-69	M	Asthma	Dyspnea, General malaise	Y	N	N
Cohort-1	Moderate	50-56	F	Diabetes, dyslipidemia	Dyspnea	N	N	N
Cohort-1	Moderate	60-69	M	-		N	N	N
Cohort-1	Moderate	50-56	M	Dilated cardiomyopathy		Y	N	N
Cohort-1	Moderate	70-79	M	-	Dyspnea	N	N	N
Cohort-1	Moderate	50-56	M	Chronic kidney disease		Y	N	N
Cohort-1	Moderate	40-49	M	Hyperuricemia	General malaise	Y	N	N
Cohort-1	Moderate	60-69	M	Hypertension, dyslipidemia, hyperuricemia	General malaise	Y	N	N
Cohort-1	Moderate	60-69	M	Hyperuricemia	General malaise	Y	N	N
Cohort-1	Moderate	60-69	M	Diabetes mellitus	General malaise	N	N	N
Cohort-1	Moderate	60-69	M	Ulcerative colitis		N	N	N
Cohort-1	Severe	80-89	M	Cholangiocarcino ma	Neurological/psychiatric symptoms	Y	N	Y
Cohort-1	Severe	60-69	M	Hypertension, hyperuricemia, dyslipidemia	General malaise	Y	Y	N

Cohort-1	Severe	60-69	M	COPD	Neurological/psychiatric symptoms, General malaise	Y	Y	N
Cohort-1	Severe	70-79	M	Cerebral infarction, gallstones	General malaise	Y	Y	N
Cohort-1	Severe	60-69	M	Hypertension, dyslipidemia	General malaise	Y	Y	Y
Cohort-1	Severe	70-79	M	Hypertension, diabetes mellitus	Dyspnea, General malaise	Y	N	Y
Cohort-1	Severe	60-69	M	-	Dyspnea, General malaise	Y	Y	N
Cohort-1	Severe	50-56	M	Post renal transplant, OSAS		Y	Y	N
Cohort-1	Severe	50-56	M	Duodenal ulcer	General malaise	Y	Y	N
Cohort-1	Severe	60-69	F	Post meningiectomy, post uterine cancer		Y	N	N
Cohort-1	Severe	80-89	M	High uric acid crystal, hypertension		Y	Y	N
Cohort-1	Severe	80-89	M	Tuberculosis, Graves' disease		Y	Y	Y
Cohort-1	Severe	70-79	M	Diabetes mellitus	Chest pain	Y	Y	N
Cohort-1	Severe	50-56	M	-	Chest pain, Dyspnea, General malaise	Y	Y	N
Cohort-1	Severe	50-56	F	Hypertension, diabetes mellitus		Y	N	N
Cohort-2	Mild	50-56	F	HIV	Headache, General malaise	-	-	-
Cohort-2	Mild	30-39	M	-	Headache, General malaise	-	-	-
Cohort-2	Mild	30-39	F	Cancer, HIV	Dyspnea, Headache, General malaise	-	-	-
Cohort-2	Mild	50-56	M	Hypertension	Coughing, Dyspnea, Headache, General malaise	Y	-	-
Cohort-2	Moderate	50-56	M	Asthma	Coughing, Headache, General malaise	-	-	-
Cohort-2	Moderate	40-49	M	-	Coughing, Headache, General malaise	-	-	-
Cohort-2	Moderate	40-49	M	COPD	General malaise	-	-	-
Cohort-2	Moderate	70-79	F	Hypertension, Atherosclerosis	Dyspnea, Chest pain, Headache	-	-	-

Cohort-2	Severe	50-56	M	Hyperlipidemia	Dyspnea, Sore throat, Fever	Y	-	-
Cohort-2	Severe	30-39	M	One kidney removed (donor)	Coughing, Fever	Y	-	-
Cohort-2	Severe	70-79	M	Inguinal Hernia	Coughing, General malaise, Fever	Y	-	-
Cohort-2	Severe	40-49	M	Appendicitis, Intestinal obstruction	Coughing, Dyspnea	Y	-	-

Supplementary Table 1. Demographic and clinical characteristics of participants. F, female; M, male; Y, yes; N, none; MPO-ANCA, myeloperoxidase-anti-neutrophil cytoplasmic antibodies; COPD, chronic obstructive pulmonary disease; OSAS, obstructive sleep apnea syndrome; HIV, human immunodeficiency virus

Name	Pearson r	p-value
Homoserine	0.703304	1.75E-08
Serine	-0.630878	1.18E-06
Aspartic acid	-0.587080	9.25E-06
Phenylalanine	-0.581179	1.19E-05
Glutamic acid	-0.558348	3.06E-05
D-Glucosamine 1-phosphate	0.487376	0.000383
Homocysteic acid	0.479218	0.000495
Glycine	-0.479059	0.000497
O-Acetyl-L-serine	-0.441200	0.001508
Carbamoyl-DL-aspartic_acid	0.438472	0.001625
3-Hydoxykynurenine	-0.432733	0.001900
Formyl-L-methionine	0.431368	0.001971
β-Hydroxybutyrate	-0.425015	0.002334
Threonine	0.415321	0.003002
Alanine	-0.412142	0.003255
4-Hydroxyproline	0.412013	0.003266
Urea	0.405406	0.003855
Tyr	0.397860	0.004640
Ethanolamine Phosphate	0.394496	0.005033
O-Phosphoserine	-0.394191	0.005070
Histidine	-0.391181	0.005448
beta-hydroxy-iso-butyrate	-0.377544	0.007487
Xanthurenic acid	0.374122	0.008093
Succinate	-0.373624	0.008184
2-oxoglutarate	-0.367412	0.009404
Asp-Asp-Ser	0.363785	0.010186
Asymmetric dimethylarginine	0.354454	0.012460
gamma-Glu-Asp	0.353619	0.012683
5-Hydroxyindole-3-acetic acid	0.349983	0.013696
Asp-Asp-Gly	0.346346	0.014776
Tryptophan	0.337811	0.017602
Ascorbic acid 2-sulfate	0.334213	0.018924
Proline	0.327451	0.021636
Dihydrooroticacid	-0.327233	0.021728

Mevalonate	0.325641	0.022414
Kynurenine	-0.323024	0.023583
Citrulline	0.316050	0.026947
Benzoic acid	-0.312753	0.028672
gamma-Glu-Citrulline	0.308060	0.031285
Ophthalmic Acid	0.306256	0.032341
gamma-Glu-His	-0.299733	0.036408
Fructose1,6-diphosphate	0.287838	0.044908
2-Hydroxyphenylacetic acid	-0.287703	0.045013
Guanosine	0.282560	0.049166
gamma-Glu-homoCys	0.282542	0.049181

Supplementary Table 2. Two-sided Pearson correlation (r) and p-values for the correlation analysis of metabolites and PCR-Ct values using COVID-19 onset initial samples (Cohort-1 samples).

Comparison	Name	AUC	p-value
Severe vs. Moderate	**Adenylsuccinic acid**	0.84524	0.00204
Severe vs. Moderate	D-Glucosamine 1-phosphate	0.83631	0.00275
Severe vs. Moderate	gamma-Glu-homoCys-Gly	0.83333	0.00415
Severe vs. Moderate	**Kyn/Trp ratio**	0.82738	0.00686
Severe vs. Moderate	Isopentenyl pyrophosphate	0.82738	0.00486
Severe vs. Moderate	p-Toluenesulfonic acid	0.82440	0.00719
Severe vs. Moderate	gamma-Glu-Arg	0.81548	0.00240
Severe vs. Moderate	gamma-Glu-homoCys	0.81250	0.01087
Severe vs. Moderate	**Kynurenine**	0.78571	0.01117
Severe vs. Moderate	Allatoin	0.78571	0.03984
Severe vs. Moderate	Cysteine	0.77976	0.00977
Severe vs. Moderate	Lanthionine	0.77679	0.04294
Severe vs. Moderate	**Threonine**	0.77381	0.01228
Severe vs. Moderate	4-Methylbenzoic acid	0.75595	0.01417
Severe vs. Moderate	trans-2-Butenoic acid	0.75595	0.01149
Severe vs. Moderate	**2-oxo-butyrate/Thr ratio**	0.75111	0.00933
Severe vs. Moderate	Cysteinesulfinic acid	0.71726	0.04989
severe vs. mild	**Adenylsuccinic acid**	1.00000	0.000003
severe vs. mild	D-Glucopyranuronate	1.00000	0.000239
severe vs. mild	D-Glucuronate	1.00000	0.000246
severe vs. mild	Cysteinesulfinic acid	1.00000	0.000000
severe vs. mild	O-Phospho-L-serine	1.00000	0.000046
severe vs. mild	2-Succinyl cysteine	1.00000	0.000003
severe vs. mild	5-Keto-gluconate	0.99405	0.000044
severe vs. mild	Cys-Gly	0.99405	0.000050
severe vs. mild	gamma-Glu-Tyr	0.98214	0.001653
severe vs. mild	**Kynurenine**	0.98214	0.000003
severe vs. mild	Quinolinate	0.97024	0.000023
severe vs. mild	Nicotinic_Acid	0.97024	0.000027
severe vs. mild	Cystine	0.97024	0.000385
severe vs. mild	Dihydrouracil	0.97024	0.000090
severe vs. mild	**Threonine**	0.97024	0.000021
severe vs. mild	Anthranilate	0.96726	0.000003
severe vs. mild	2,6-Pyridinedicarboxylic acid	0.96429	0.000025

severe vs. mild	O-Acetyl-L-serine	0.96429	0.000004
severe vs. mild	gamma-Glu-Trp	0.96131	0.000196
severe vs. mild	4-Hydroxyphenyllactic acid	0.95833	0.000022
severe vs. mild	2-oxoglutarate	0.95833	0.000024
severe vs. mild	Cystathionine	0.95833	0.000007
severe vs. mild	**Kyn/Trp ratio**	0.95294	0.000002
severe vs. mild	Homovanillic acid	0.95238	0.000022
severe vs. mild	4-Methylbenzoic acid	0.94643	0.000034
severe vs. mild	**Kynurenine**	0.94345	0.000096
severe vs. mild	Homocystine	0.94345	0.000002
severe vs. mild	Thiosulfate	0.94048	0.000011
severe vs. mild	gamma-Glu-Glu	0.94048	0.001688
severe vs. mild	gamma-Glu-Val	0.93452	0.000364
severe vs. mild	Phenylalanine	0.92857	0.000001
severe vs. mild	Phenylpyruvic acid	0.92857	0.000058
severe vs. mild	**2-oxo-butyrate**	0.92857	0.000317
severe vs. asymptomatic	**Adenylsuccinic acid**	1.00000	0.00027
severe vs. asymptomatic	**Kynurenine**	1.00000	0.00021
severe vs. asymptomatic	**Kyn/Trp ratio**	1.00000	0.00020
severe vs. asymptomatic	5-Keto-gluconate	1.00000	0.01127
severe vs. asymptomatic	Cytosine	1.00000	0.00019
severe vs. asymptomatic	L-Cysteinesulfinic acid	1.00000	0.00095
severe vs. asymptomatic	Cys-Gly	1.00000	0.00947
severe vs. asymptomatic	2,6-Pyridinedicarboxylic acid	0.98611	0.00307
severe vs. asymptomatic	D-Glucopyranuronate	0.98611	0.03080
severe vs. asymptomatic	Quinolinate	0.98611	0.00285
severe vs. asymptomatic	D-Glucuronate	0.98611	0.03086
severe vs. asymptomatic	Nicotinic_Acid	0.98611	0.00345
severe vs. asymptomatic	O-Phospho-L-serine	0.98611	0.00342
severe vs. asymptomatic	p-Toluenesulfonic acid	0.97222	0.00059
severe vs. asymptomatic	trans-2-Butenoic acid	0.97222	0.04218

severe vs. asymptomatic	Dihydrouracil	0.97222	0.00613
severe vs. asymptomatic	Urocanic acid	0.97222	0.01848
severe vs. asymptomatic	N-Acetyl-D-glucosamine	0.96528	0.00883
severe vs. asymptomatic	**Threonine**	0.95833	0.00079
severe vs. asymptomatic	**Tryptophan**	0.95833	0.00100
severe vs. asymptomatic	Phenylalanine	0.94444	0.00081
severe vs. asymptomatic	3-Hydoxykynurenine	0.94444	0.00578
severe vs. asymptomatic	2-oxoglutarate	0.94444	0.00736
severe vs. asymptomatic	PLP	0.94444	0.00038
severe vs. asymptomatic	Thiosulfate	0.94444	0.00071
severe vs. asymptomatic	Anthranilate	0.94444	0.00225
severe vs. asymptomatic	Glutamine	0.93056	0.00176
severe vs. asymptomatic	Cytidine	0.93056	0.02359
severe vs. asymptomatic	gamma-Glu-homoCys	0.93056	0.00638
severe vs. asymptomatic	gamma-Glu-homoCys-Gly	0.93056	0.08971
severe vs. asymptomatic	N-Methyl-Arg	0.93056	0.00158
severe vs. asymptomatic	Kynurenic acid	0.91667	0.05936

Supplementary Table 3. AUC and p-values for ROC curve analysis of metabolites that could predictably discriminate COVID-19 severe pneumonia patients from moderate, mild, and asymptomatic groups.
Metabolites in bold represent amino acids and their catabolites that are the top candidates for discriminative markers of severe disease.

Name	f-value	p-value	-LOG10 (p)	FDR
CXCL10 / P-10 / CRG-2	13.987	3.20E-08	7.4954	7.03E-07
IL-4	6.9878	1.01E-04	3.9946	8.55E-04
IL-1a / IL-1F1	6.8791	1.17E-04	3.9334	8.55E-04
IL-1ra / IL-1F3	6.6485	1.58E-04	3.8026	8.67E-04
IL-10	4.8329	0.0018321	2.7371	0.008061
CXCL9 / MIG	3.9765	0.0061167	2.2135	0.015621
IL-2	3.9508	0.0063451	2.1976	0.015621
IFN-a	3.9458	0.0063905	2.1945	0.015621

Supplementary Table 4. Prognostic performance of cytokines in Cohort-1 serum samples for discrimination of severe and moderate disease outcomes by ROC curve analysis

	Gene	Forward	Reverse
Mouse	*Ifng*	GCCACGGCACAGTCATTGA	TGCTGATGGCCTGATTGTCTT
	Cxcl10	CCAAGTGCTGCCGTCATTTTC	TCCCTATGGCCCTCATTCTCA
	Cxcl9	GGAGTTCGAGGAACCCTAGTG	GGGATTTGTAGTGGATCGTGC
	Il6	TGATGCACTTGCAGAAAACA	ACCAGAGGAAATTTTCAATAGGC
	Il10	CAGAGCCACATGCTCCTAGA	TGTCCAGCTGGTCCTTTGTT
	Ido1	CAAAGCAATCCCCACTGTATCC	ACAAAGTCACGCATCCTCTTAAA
	Ido2	CCAGAAGGACCGTTGGAAATC	ACTGTCACTAGGATGAAGCCC
	Bcat1	GAAGTGGCGGAGACTTTTAGG	TGGTCAGTAAACGTAGCTCCA
	Bcat2	AAAGCATACAAAGGTGGAGACC	CGTAGAGGCTCGTTCCGTTG
	Mki67	ATCATTGACCGCTCCTTTAGGT	GCTCGCCTTGATGGTTCCT
	Rn18s	GCAATTATTCCCCATGAACG	GGCCTCACTAAACCATCCAA
	ActB	GATCTGGCACCACACCTTCT	GGGGTGTTGAAGGTCTCAAA

Supplementary Table 5. List of primers for qPCR.

References

1. Zhang, P. et al. Vitamin B6 Prevents IL-1β Protein Production by Inhibiting NLRP3 Inflammasome Activation. *Journal of Biological Chemistry* **291**, 24517-24527 (2016).
2. Macdonald, R. Red cell 2,3-diphosphoglycerate and oxygen affinity. *Anaesthesia* **32**, 544-553 (1977).
