# Supplementary material for: Amino acid catabolite markers for early prognostication of pneumonia in patients with COVID-19
Source: Nat Commun. 2023 Dec 20;14:8469. doi: 10.1038/s41467-023-44266-z (PMC10733290; doi:10.1038/s41467-023-44266-z)
Supplement: Supplementary file 3 — Description of Additional Supplementary Files [file 41467_2023_44266_MOESM3_ESM.pdf]

File Name: Supplementary Data 1
Description: Supplementary data 1 is the quantitative data for water-soluble metabolites obtained by IC-HR-MS and LC-MS/MS

File Name: Supplementary Data 2
Description: Supplementary data 2 is the quantitative data for lipidome analysis obtained by LC-HR-MS.

File Name: Supplementary Data 3
Description: Supplementary data 3 shows quantitative data for steroids obtained by LC-MS/MS.

File Name: Supplementary Data 4
Description: The MRM library of compounds measured by LC-MS/MS is available as Supplementary data 4.
