# Supplementary material for: Amino acid catabolite markers for early prognostication of pneumonia in patients with COVID-19
Source: Nat Commun. 2023 Dec 20;14:8469. doi: 10.1038/s41467-023-44266-z (PMC10733290; doi:10.1038/s41467-023-44266-z)
Supplement: Supplementary file 8 — Reporting Summary [file 41467_2023_44266_MOESM8_ESM.pdf]

nature portfolio

Corresponding author(s): Yuki Sugiura

Last updated by author(s): 23/11/23

Reporting Summary

Nature Portfolio wishes to improve the reproducibility of the work that we publish. This form provides structure for consistency and transparency in reporting. For further information on Nature Portfolio policies, see our Editorial Policies and the Editorial Policy Checklist.

Statistics

For all statistical analyses, confirm that the following items are present in the figure legend, table legend, main text, or Methods section.

n/a	Confirmed	
☐	☒	The exact sample size (*n*) for each experimental group/condition, given as a discrete number and unit of measurement
☐	☒	A statement on whether measurements were taken from distinct samples or whether the same sample was measured repeatedly
☐	☒	The statistical test(s) used AND whether they are one- or two-sided<br>*Only common tests should be described solely by name; describe more complex techniques in the Methods section.*
☒	☐	A description of all covariates tested
☐	☒	A description of any assumptions or corrections, such as tests of normality and adjustment for multiple comparisons
☐	☒	A full description of the statistical parameters including central tendency (e.g. means) or other basic estimates (e.g. regression coefficient) AND variation (e.g. standard deviation) or associated estimates of uncertainty (e.g. confidence intervals)
☐	☒	For null hypothesis testing, the test statistic (e.g. *F*, *t*, *r*) with confidence intervals, effect sizes, degrees of freedom and *P* value noted<br>*Give P values as exact values whenever suitable.*
☒	☐	For Bayesian analysis, information on the choice of priors and Markov chain Monte Carlo settings
☒	☐	For hierarchical and complex designs, identification of the appropriate level for tests and full reporting of outcomes
☐	☒	Estimates of effect sizes (e.g. Cohen's *d*, Pearson's *r*), indicating how they were calculated

Our web collection on statistics for biologists contains articles on many of the points above.

Software and code

Policy information about availability of computer code

Data collection

The following software was used for metabolome and imaging MS measurements;
- Nexera LC system operating software (Shimadzu Co., Kyoto, Japan).
- High-performance bench-top quadrapole Orbitrap mass spectrometer operating software (Q Exactive focus, Thermo Fisher Scientific, MA, USA).
- Xcalibur 4.2.47 software (Thermo Fisher Scientific, MA, USA).
- Lab Solutions (ver. 5.113, Shimadzu)
- MALDI-TOF MS operating software (timsTOF flex, Bruker Daltonics, Bremen, Germany).

Data analysis

The following software was used for metabolome and imaging MS data analysis;
- Trace Finder software (v 3.1, Thermo Fisher Scientific)
- Scils Lab software (Bruker Daltonics)
- Graph-pad Prism 9.5.1 was used for data presentation and statistical analysis.

For manuscripts utilizing custom algorithms or software that are central to the research but not yet described in published literature, software must be made available to editors and reviewers. We strongly encourage code deposition in a community repository (e.g. GitHub). See the Nature Portfolio guidelines for submitting code & software for further information.

Data

Policy information about availability of data

All manuscripts must include a data availability statement. This statement should provide the following information, where applicable:

- Accession codes, unique identifiers, or web links for publicly available datasets
- A description of any restrictions on data availability
- For clinical datasets or third party data, please ensure that the statement adheres to our policy

The authors declare that the data generated or analyzed in this study are available from the corresponding author upon reasonable request.

Research involving human participants, their data, or biological material

Policy information about studies with human participants or human data. See also policy information about sex, gender (identity/presentation), and sexual orientation and race, ethnicity and racism.

Reporting on sex and gender	Sex information was collected for SARS-CoV-2 infected patients but not for uninfected participants (Table 1). No subgroup analyses on sex were performed due to small sample size.
Reporting on race, ethnicity, or other socially relevant groupings	Information about the individuals enrolled in this study is shown in Table 1: age range, sex, disease state, and diagnosis and treatment.
Population characteristics	The demographic and clinical characteristics of the participants are shown in Table 1.
Recruitment	In this study, residual serum samples for biochemical and immunologic tests were collected from patients with suspected SARS-CoV-2 infection who underwent RT-PCR testing of nasopharyngeal swabs or saliva samples at Keio University Hospital or Osaka Metropolitan University Hospital from March 2020 to January 2021.
Ethics oversight	Sample collection and utilization were conducted under the approval of the Ethics Committee of the Keio University School of Medicine (approval numbers 20200059 and 20200063) and the Ethics Committee of Osaka Metropolitan University Graduate School of Medicine (approval number 2020-003).

Note that full information on the approval of the study protocol must also be provided in the manuscript.

Field-specific reporting

Please select the one below that is the best fit for your research. If you are not sure, read the appropriate sections before making your selection.

☒ Life sciences ☐ Behavioural & social sciences ☐ Ecological, evolutionary & environmental sciences

For a reference copy of the document with all sections, see nature.com/documents/nr-reporting-summary-flat.pdf

Life sciences study design

All studies must disclose on these points even when the disclosure is negative.

Sample size	Sample size calculations were not performed. The number of human samples was determined based on availability, and the number of animals was determined based on the number of animals used in previously published work. Each experiment was replicated for subsequent statistical analysis.
Data exclusions	No data were excluded from the results obtained in this study.
Replication	As indicated in the figures, figure legends and methods, each animal experiment was performed with at least 3 biological replicates. Each human clinical trial was performed with at least 3 biological replicates. All replication attempts yielded similar results and were reliably reproduced.
Randomization	All samples and animals were randomly allocated into experimental groups.
Blinding	No blinded testing was performed because the same investigator set up the experiments, collected the samples, and analyzed the data. However, the samples and data were collected under identical conditions.

Reporting for specific materials, systems and methods

We require information from authors about some types of materials, experimental systems and methods used in many studies. Here, indicate whether each material, system or method listed is relevant to your study. If you are not sure if a list item applies to your research, read the appropriate section before selecting a response.

Materials & experimental systems

n/a	Involved in the study
☐	☒ Antibodies
☒	☐ Eukaryotic cell lines
☒	☐ Palaeontology and archaeology
☐	☒ Animals and other organisms
☒	☐ Clinical data
☒	☐ Dual use research of concern
☒	☐ Plants

Methods

n/a	Involved in the study
☒	☐ ChIP-seq
☒	☐ Flow cytometry
☒	☐ MRI-based neuroimaging

Antibodies

Antibodies used

Monoclonal antibodies specific to human IL-28B/IFN-l3 included in Human IL-28B/IFN-lambda 3 Quantikine ELISA Kit (R&D Systems, catalog #D28B00)
Primary antibodies for immunostaining; Prosurfactant Protein C Antibody (Novus, CO, US, catalog #H00006440-M01, clone 4A10, dilution: 1:1000) and CXCL10 Polyclonal Antibody (Bioss, MA, US, catalog #BS-1502R, dilution: 1:500)
Secondary antibodies for immunostaining; Alexa Fluor 647 (anti-Armenian hamster, IgG, Jackson Immuno Research, PA, US) and Alexa Fluor 488 (anti-Rabbit, IgG, abcam Cambridge, UK)

Validation

All antibodies and kits came from commercial vendors, and were validated by the manufacturers on their websites.
Primary antibodies
Prosurfactant Protein C Antibody; Data sheet; https://www.novusbio.com/PDFs/H00006440-M01.pdf
CXCL10 Polyclonal Antibody; Data sheet; https://www.biossantibodies.com/datasheets/bs-1502R

Animals and other research organisms

Policy information about studies involving animals; ARRIVE guidelines recommended for reporting animal research, and Sex and Gender in Research

Laboratory animals

Mouse (BALB/cCrSlc and C57BL/6 NCrSlc for SARS-CoV-2 infection model, and C57BL/6J for Influenza virus infection model).

Wild animals

This study did not use wild animals.

Reporting on sex

Female mice were used for all experiments in this study.

Field-collected samples

This study did not include samples from field-collected samples.

Ethics oversight

All experiments were conducted in accordance with protocols approved by the Institutional Animal Care and Use Committee of the Keio University and Osaka University.

Note that full information on the approval of the study protocol must also be provided in the manuscript.
